# Weight loss by sodium-glucose co-transporter 2 inhibitor canagliflozin is followed by rebound in *db/db* mice but maintained in fat-fed mice via circadian rises in body temperature and locomotor activity

**DOI:** 10.1016/j.jphyss.2026.100079

**Published:** 2026-05-14

**Authors:** Lei Wang, Wanxin Han, Dauren Zhantleu, Seiya Banno, Yermek Rakhat, Nazymgul Kulzhanova, Abilkhair Yussupov, Yusaku Iwasaki, Masanori Nakata, Daisuke Yabe, Yutaka Seino, Toshihiko Yada

**Affiliations:** a Center for Integrative Physiology, Kansai Electric Power Medical Research Institute, Osaka 553-0003, Japan; b Department of Diabetes, Endocrinology and Metabolism/Rheumatology and Clinical Immunology, Gifu University Graduate School of Medicine, Gifu 501-1194, Japan; c Division of Diabetes, Metabolism and Endocrinology, Kobe University Graduate School of Medicine, Kobe 650-0047, Japan; d Division of Health Sciences, Medicine and Aging, Bin Zhou Polytechnic, Bin Zhou 256-603, China; e Department of Endocrinology, Diabetes and Metabolism, Fujita Health University School of Medicine, Aichi 470-1192, Japan; f Laboratory of Animal Science, Graduate School of Life and Environmental Sciences, Kyoto Prefectural University, Kyoto 606-8522, Japan; g Department of Physiology, School of Medicine, Wakayama Medical University, Wakayama 641-8509, Japan; h Yutaka Seino Distinguished Center for Diabetes Research, Kansai Electric Power Hospital, Osaka 553-0003, Japan; i Departments of Diabetes, Endocrinology and Nutrition, Kyoto University Graduate School of Medicine, Kyoto 606-8507, Japan; j Center for One Medicine Innovative Translational Research, Gifu University Institute for Advanced Study, Gifu, Japan

**Keywords:** SGLT2 inhibitor, Energy balance, Circadian rhythm, Weight gain, Body temperature, Locomotor activity

## Abstract

Sodium-glucose co-transporter 2 (SGLT2) inhibitors, diabetic medicines, induce glucosuria to lower glycemia and energy reserve, initially reducing weight. The weight-reducing capacity substantially differs between individuals, likely due to both social/psychological factors and physical/metabolic states. Exclusively analyzing the contribution of physical/metabolic states is difficult in humans but feasible in mice. To explore whether metabolic states can alter weight outcomes of SGLT2 inhibitor treatment, the impact of canagliflozin was studied comparatively in high-fat-diet-induced obese (DIO) mice and leptin-receptor-deficient *db/db* mice. Canagliflozin, orally administered, induced rapid reductions in glycemia and weight followed by elevated food intake in both models. The initial weight loss was rebounded in *db/db* mice, but progressed and sustained in DIO mice accompanied by circadian rises in body temperature and locomotor activity, the effects possibly balancing the elevated food intake. These results reveal that physical/metabolic states, independent of social/psychological factors, influence weight outcome of canagliflozin treatment.

## Graphical Abstract

**Figure:**
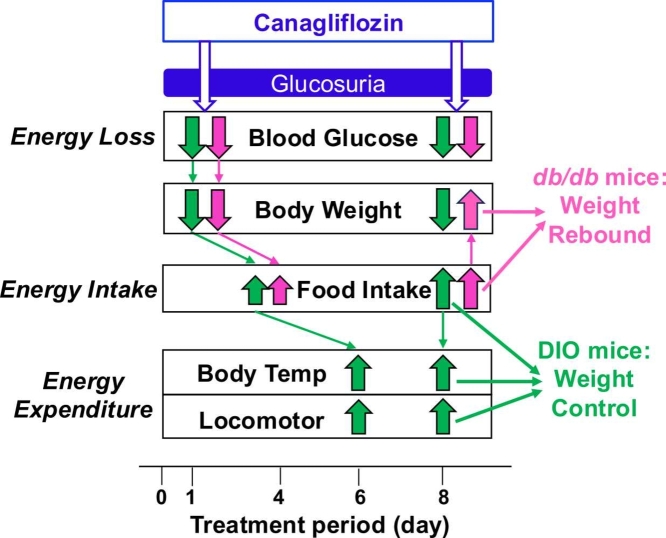

## Introduction

Obesity is a growing health issue worldwide. People with obesity are predisposed to a variety of diseases including type 2 diabetes, dyslipidemia, cardiovascular diseases, kidney diseases, and cancer. Effective and safe medicines to treat obesity and related diseases have long been awaited. Sodium-glucose co-transporter-2 (SGLT2) inhibitors are currently licensed for the treatment of type 2 diabetes, and a growing body of evidence supports their use for cardiovascular and renal diseases [1], [2], [3], [4]. These drugs block the SGLT2 protein in the proximal convoluted tubule and thereby enhance glucosuria. This action is associated with an increased urinary loss of approximately 60–100 g of glucose (200–300 kcal) per day [5], which can trigger a reduction in body weight [6]. The ability to reduce body weight is consistently observed in individuals taking SGLT2 inhibitors.

However, the body weight reduction is often moderate and varies substantially between individuals [6], [7], [8]. Furthermore, the initial body weight loss continues in some individuals but diminishes in others [9]. The individual difference could derive from the two components, physical/metabolic states and social/psychological factors [10], [11], [12], [13]: it is difficult to clearly separate the influence of one from the influence of the other between the two components in humans. Hence, the contribution of each component is not well understood.

The aim of the present study is to explore whether physical/metabolic states, independent of social/psychological factors, can determine the outcome of therapy with SGLT2 inhibitors. For this, the use of mouse models appears a good approach since social/psychological factors such as motivation for therapy do not apply. This study explores the effect of a SGLT2 inhibitor, canagliflozin, on body weight in two mouse models with distinct metabolic states, high-fat diet-induced obese (DIO) mice and leptin receptor-mutated diabetic obese *db/db* mice: these models with almost identical body weight were used to exclude the influence of degree of obesity. Another issue of interest is how the body responds to the energy loss (glucosuria) by altering energy intake and/or expenditure. To address it, food intake, body temperature and locomotor activity were measured. This study was conducted only in male mice based on the documents that the attainment of treatment goals on body weight and hemoglobin A1c with canagliflozin 100 or 300 mg for 26 weeks was similar in both genders [14] and that the outcomes in SGLT2 inhibitor treatment were sex-consistent [15], [16].

## Materials and methods

### Materials

Canagliflozin was provided by Tanabe Pharma Corporation (Tokyo, Japan). Hydroxypropyl methylcellulose （HPMC） was purchased from Wako (Tokyo, Japan).

### Animals

**Fig. 1:**
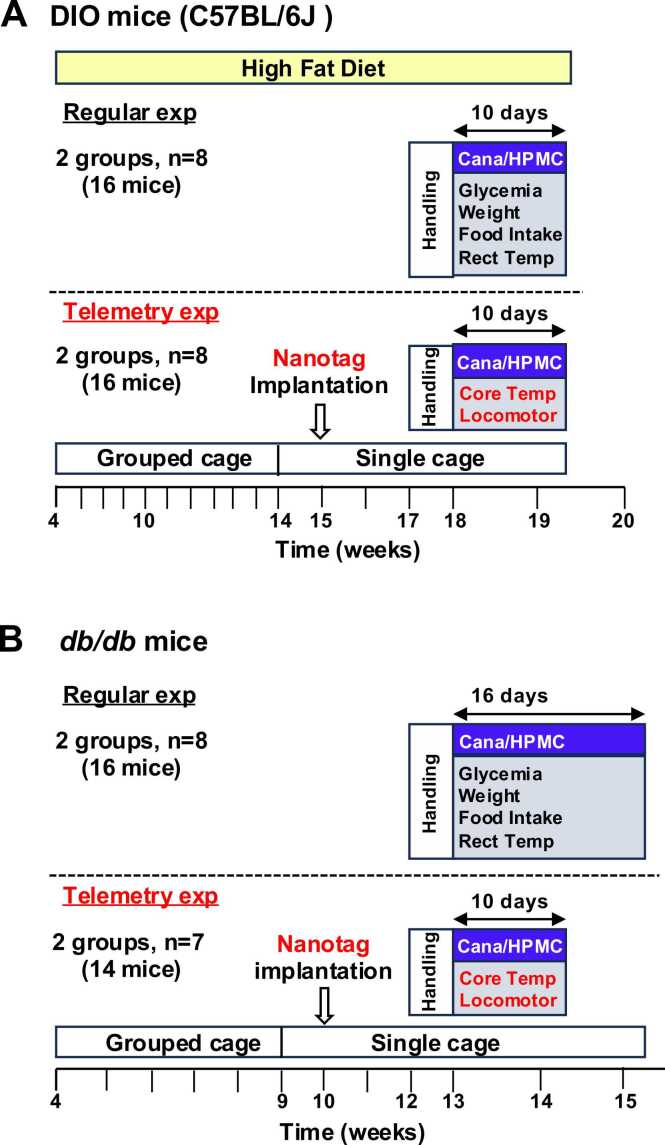
Schema of study time-line**.** Protocols for regular experiments (upper panels) and telemetry experiments (lower panels) in DIO mice (**A**) and *db/db* mice (**B**). Time-line of HFD feeding, shift from grouped to single cages, nanotag implantation, handling, oral gavage of drug, and measurement of metabolic parameters are illustrated.

Only male mice were used in this study. C57BL/6 J mice were purchased from CLEA Japan Inc. (Tokyo, Japan) and fed the high-fat diet D12492 containing 60% fat, 20% protein, and 20% carbohydrate (Research Diets Inc.; New Brunswick, NJ, USA) from 4 weeks old to yield diet-induced obese (DIO) mice (Fig. 1A). The DIO mice were housed in grouped cages (4 mice), moved to single cages at 14 weeks, and subjected to handling from 17 weeks for a week, followed by administration of canagliflozin or HPMC (control) by oral gavage from 18 weeks (Fig. 1A). The 2 groups, each composed of 8 DIO mice, were used for regular experiments and for telemetry experiments. In telemetry experiments, nanotag was implanted at 15 weeks (Fig. 1A). C57BLKS/J Iar -+Leprdb/+Leprdb (*db/db*) mice were purchased from CLEA Japan Inc. (Tokyo, Japan) and fed standard chow CE-2 (CLEA Japan Inc.). The *db/db* mice were housed in grouped cages, moved to single cages at 9 weeks, and subjected to handling from 12 weeks, followed by administration of canagliflozin or HPMC by oral gavage from 13 weeks (Fig. 1B). The 2 groups, each composed of 8 *db/db* mice, were used for regular experiments, and 2 groups, each composed of 7 *db/db* mice, were used for telemetry experiments. In telemetry experiments, nanotag was implanted at 10 weeks (Fig. 1B). All mice were housed under conditions of controlled temperature (23 ± 1°C) and humidity (55 ± 5%) with a 12 h light/12 h dark cycle (lights on at 08:00) and free access to food and water. The animal experiments were carried out after receiving approval from the Institutional Animal Experiment Committee and in accordance with the Institutional Regulation for Animal Experiments at Kobe University (approval number: 30–10–06-R1) and Gifu University (IACUC approval number: 2021–235).

### Drug administration and measurement of blood glucose, food intake, body weight, and water intake

All mice housed in individual cages were subjected to handling for 1 week before experiments. Either canagliflozin at 30 mg/kg body weight, the dose selected from previous studies [17], [18], [19], [20], or hydroxypropyl methyl cellulose (HPMC) aqueous solvent (0.5%) as the control was administered to DIO mice or *db/db* mice at 10:00 once daily by oral gavage, following the route of SGLT2 inhibitors administration in humans. Just before the administration at 10:00, blood glucose, water intake, body weight, and food intake were measured for 10 days (Fig. 1A,B). The period was extended to 16 days in regular experiments in *db/db* mice (Fig. 1B), since body weight was increasing, but not stabilized, on day 10.

Blood samples were collected from the tail vein using heparinized capillary glass tubes. Plasma was collected after centrifugation (4000 rpm, 10 min at 4°C) and stored at −80°C until analysis. Blood glucose levels were detected using a GLUCOCARD Plus Care GT-1840 (ARKRAY Factory Inc.; Koka, Shiga, Japan). Body weight was measured using Electronic Balance EntrisR II BCE Essential Series (Sartorius AG; Gettingen, Germany). To measure daily food intake and water intake under free access to food and water, the food in feedbox and water in water bottle were carefully weighted at 10:00 on day N and day N + 1, and the difference in the values was considered the daily food intake and daily water intake on day N + 1.

### Measurement of rectal temperature

To measure rectal temperature, the mice were held still by holding the skin on the neck and back, and a temperature probe was inserted into the anus until a stable reading was obtained using a BAT-12 digital thermometer (Physitemp Instruments; Clifton, NJ, USA).

### Telemetry using nanotag for monitoring body core temperature and locomotor activity

The body core temperature and locomotor activity in free-moving mice were measured sequentially with nano tag® system (Kissei Comtec Co., Ltd.; Matsumoto, Japan) [21], combined with a nanotag viewer software and PaSoRi radio frequency identification reader (SONY Co., Ltd.; Tokyo, Japan). The nanotag device has a temperature sensor to detect body core temperature and a 3-axis capacitive accelerometer that detects activity count, but not cross count (http://www.sleepsign.com/nanotag/spec.html) [22].

### Implantation of nanotag

Mice were moved to single cages for 1 week for environmental adaptation before nanotag insertion (Fig. 1A,B). Nanotag devices were implanted as previously reported [21], [22], [23]. Briefly, nanotag was set to automatically record body temperature and locomotor activity at 5 min intervals. Mice were anesthetized using 2% isoflurane, the skin was cut longitudinally along the midline of abdomen to expose the inner side of abdominal cavity, and nanotag was inserted between the abdominal cavity and gastrointestinal tract and secured to the extramuscular membrane using nylon threads. Then skin was sutured. No complications or severe infections were observed. The mice recovered from the reduced food intake due to operation within 2 weeks, followed by handling and oral gavage of canagliflozin/HPMC (Fig. 1A,B). The body temperature and locomotion activity of the mice were measured every 5 min for 10 days, followed by collection of recorded data.

### Statistical analysis

Data are presented as mean ± SEM. Assumptions for parametric tests were verified. Statistical analysis was performed using two-way ANOVA followed by Sidak's multiple comparisons test and two-tailed unpaired *t*-test between two groups. All statistical analyses were performed using Prism 8 (GraphPad Software; Boston, MA, USA). P < 0.05 was considered significant. More specific and detailed statistics are described in the corresponding Figure legends.

## Results

### Effects of canagliflozin on blood glucose, water intake, body weight, and food intake in DIO mice

**Fig. 2:**
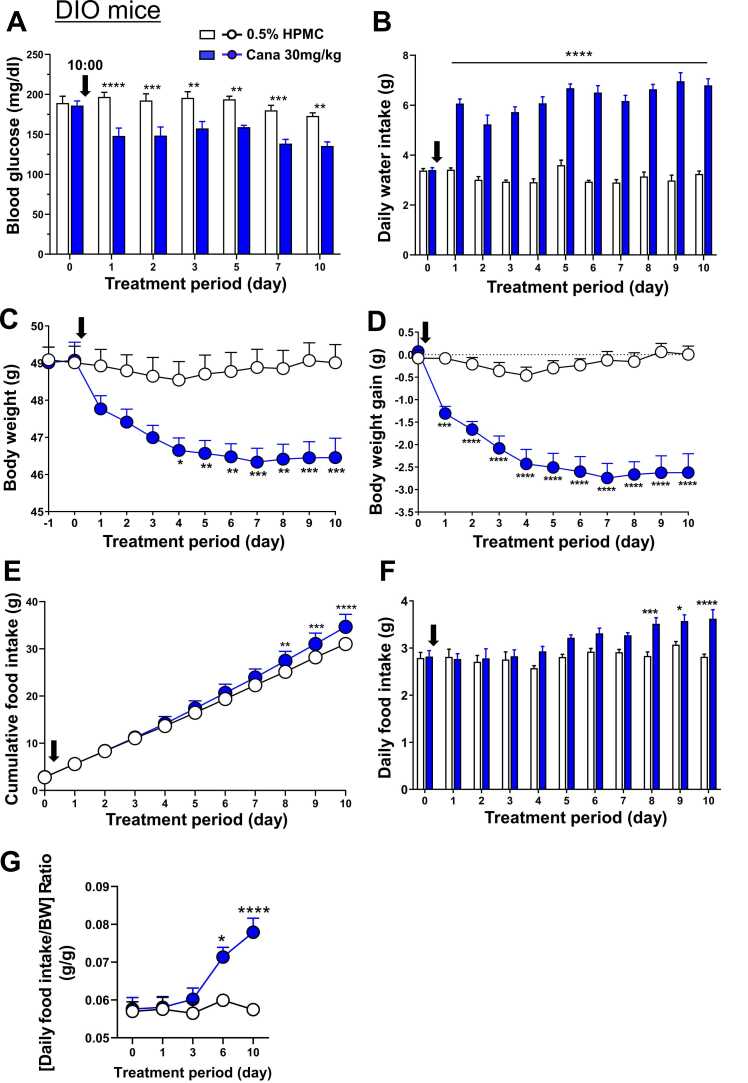
Effects of canagliflozin on blood glucose, water intake, body weight, and food intake in DIO mice. Canagliflozin was administered by oral gavage to DIO mice aged 18 weeks at 10:00 once daily for 10 days. Vertical arrows indicate the timing of the first injection. **A**: Canagliflozin significantly decreased blood glucose from day 1 to day 10. **B**: Canagliflozin significantly increased daily water intake from day 1 to day 10. **C-D**: Canagliflozin significantly decreased body weight on days 4–10 (C) and body weight gain on days 1–10 (D). **E-F**: Canagliflozin tended to increase cumulative food intake (E) and daily food intake (F) on days 4–7, and significantly increased food intake (E) and daily food intake (F) on days 8–10. **G**: Canagliflozin significantly increased [daily food intake/body weight (BW)] ratio on day 6 and day 10. n = 8 in each group. All data are presented as mean ± SEM. *P < 0.05, **P < 0.01, ***P < 0.001, and ****P < 0.0001 according to two-way ANOVA followed by Sidak's multiple comparisons test.

Canagliflozin at 30 mg/kg suspended in 0.5% HPMC and control 0.5% HPMC were administered by oral gavage to DIO mice (weight, around 49 g) at 10:00 once daily for 10 days. On day 1 of the canagliflozin treatment, compared to the control, blood glucose significantly decreased (Fig. 2A) and water intake significantly increased (Fig. 2B). These changes on day 1 continued through day 10, the end of the treatment. Body weight significantly decreased on day 4 and later (Fig. 2C). Body weight gain was significantly reduced on day 1, and this reduction was progressed until day 6 and then sustained on days 7–10 (Fig. 2D). Canagliflozin tended to increase cumulative food intake (Fig. 2E) and daily food intake (Fig. 2F) from day 4, reaching significant increases on days 8–10. The [daily food intake/body weight] ratio significantly increased on day 6 and day 10 (Fig. 2G).

### Effects of canagliflozin on blood glucose, water intake, body weight, and food intake in db/db mice

**Fig. 3:**
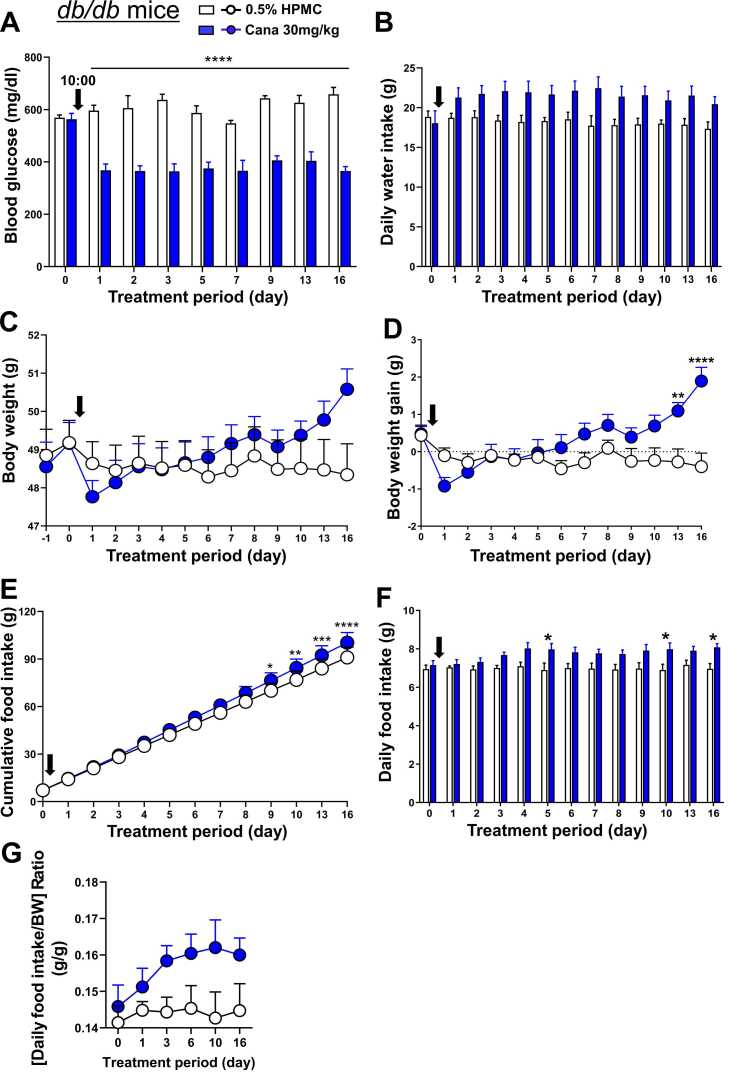
Effects of canagliflozin on blood glucose, water intake, body weight, and food intake in *db/db* mice**.** Canagliflozin was administered by oral gavage to *db/db* mice aged 13 weeks at 10:00 for 10 days. Vertical arrows indicate the timing of the first injection. **A**: Canagliflozin significantly decreased blood glucose from day 1 to day 16. **B**: Canagliflozin tended to increase daily water intake from day 1 to day 16. **C-D**: Under canagliflozin treatment, body weight (C) and body weight gain (D) tended to decrease on days 1–2 and increase on days 6–10, reaching significant increase in body weight gain on days 13 and 16 (D). **E-F**: Canagliflozin tended to increase cumulative food intake (E) and daily food intake (F) from day 4, reaching significant increases in cumulative food intake on days 9–16 (E) and daily food intake on days 5, 10, and 16 (F). **G**: Effect of canagliflozin on [daily food intake/body weight (BW)] ratio. n = 8. All data are presented as mean ± SEM. *P < 0.05, **P < 0.01, ***P < 0.001, and ****P < 0.0001 according to two-way ANOVA followed by Sidak's multiple comparisons test.

Canagliflozin at 30 mg/kg was administered by oral gavage to *db/db* mice (weight, around 49 g) under the same conditions as in DIO mice for 16 days. On day 1 of canagliflozin treatment, compared to 0.5% HPMC (control), blood glucose significantly decreased (Fig. 3A) and water intake tended to increase (Fig. 3B). These changes continued through day 16.

The body weight and body weight gain in canagliflozin-treated mice, compared to control, showed tendency of decrease on days 1–2 (Fig. 3C,D) and increase on days 6–16. The body weight gain increased progressively, reaching significant levels on days 13 and 16. Canagliflozin significantly increased cumulative food intake on days 9, 10, 13, and 16 (Fig. 3E) and daily food intake on days 5, 10, and 16 (Fig. 3F). The [daily food intake/body weight] ratio appeared to be higher in canagliflozin group than control group from day 3 through day 16 of treatment (Fig. 3G).

### Effects of canagliflozin on body temperature and locomotor activity in DIO and db/db mice

**Fig. 4:**
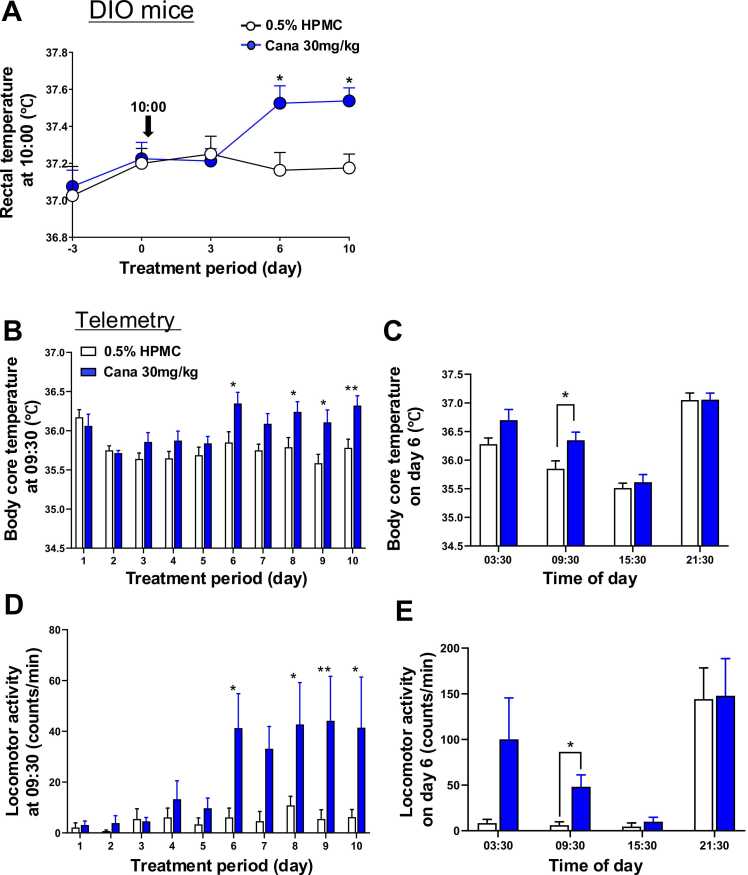
Effects of canagliflozin on body temperature and locomotor activity in DIO mice**.** Canagliflozin was administered by oral gavage to DIO mice aged 18 weeks at 10:00 for 10 days. **A**: Canagliflozin significantly increased rectal temperature on day 6 and day 10. **B-E**: Results of telemetry with nanotag. **B**: Canagliflozin increased body core temperature on days 6, 8, 9, and 10. **C**: Canagliflozin increased body core temperature at 09:30 but not at 03:30, 15:30, or 21:30. **D**: Canagliflozin increased locomotor activity on days 6, 8, 9, and 10. **E**: Canagliflozin increased locomotor activity at 09:30 but not at 03:30, 15:30, or 21:30. n = 8. All data are presented as mean ± SEM. *P < 0.05, **P < 0.01, and ****P < 0.0001 according to two-way ANOVA followed by Sidak's multiple comparisons test.

In DIO mice, canagliflozin elevated rectal temperature on day 6 and day 10 of treatment (Fig. 4A). This time course coincided with that of increased [daily food intake/body weight] (Fig. 2G). To expand the outcomes, we recorded the body core temperature continuously using a telemetry system with an implanted nanotag sensor. In telemetry experiments, canagliflozin elevated body core temperature on days 6, 8, 9, and 10 of treatment (Fig. 4B). Considering that body temperature exhibits circadian rhythm [24], [25], we measured body core temperature on several time points. In the control mice, body temperature was higher at 21:30 and 03:30 in the active phase, and lower at 09:30 and 15:30 in the resting phase, showing circadian changes (Fig. 4C). Canagliflozin significantly elevated body core temperature at 09:30 by counteracting its circadian lowering on day 6 (Fig. 4C). Next, locomotor activity, which is also known to exhibit circadian rhythm [26], was analyzed. Canagliflozin elevated locomotor activity at 09:30 on days 6, 8, 9, and 10 of treatment (Fig. 4D). In the control mice, locomotor activity was high at 21:30, the early active phase, and low at 03:30, 09:30, and 15:30, showing circadian rhythm (Fig. 4E). Canagliflozin significantly elevated locomotor activity at 09:30 on day 6 (Fig. 4E).

**Fig. 5:**
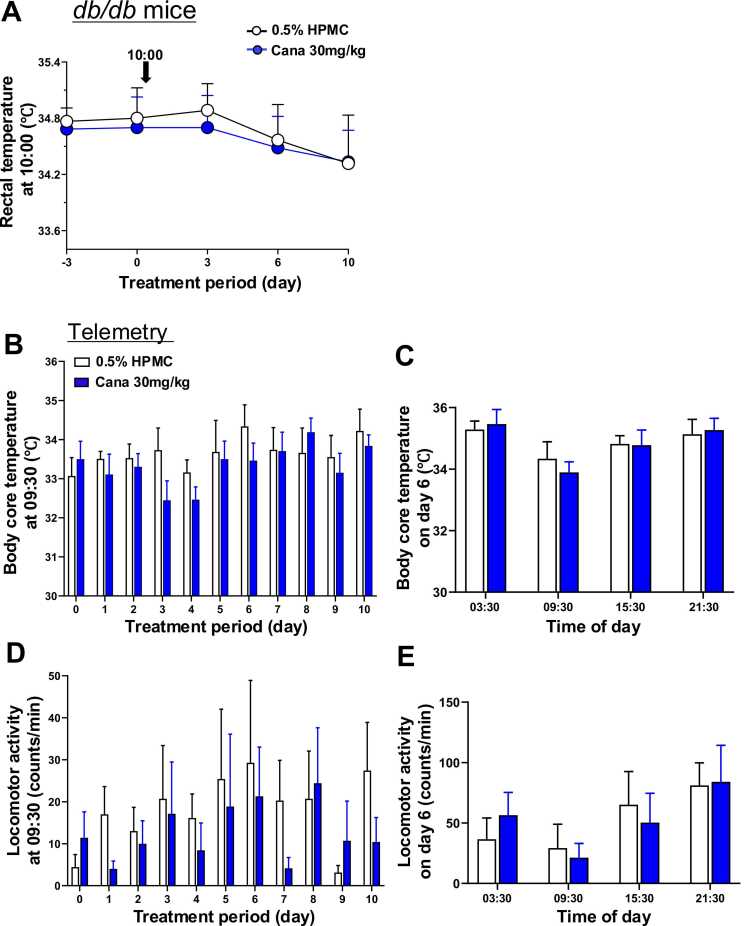
Effects of canagliflozin on body temperature and locomotor activity in *db/db* mice**.** Canagliflozin was administered by oral gavage to DIO mice aged 13 weeks at 10:00 for 10 days. **A**: Canagliflozin had no effect on rectal temperature on days 3, 6 and 10. **B-E**: Results of telemetry with nanotag. **B**: Canagliflozin had no effect on body core temperature from day 1 to day 10. **C**: Canagliflozin had no effect on body core temperature at 03:30, 09:30, 15:30 and 21:30 on day 6. **D**: Canagliflozin had no effect on locomotor activity from day 1 to day 10. **E**: Canagliflozin had no effect on locomotor activity at 03:30, 09:30, 15:30 and 21:30 on day 6. n = 7–8. All data are presented as mean ± SEM.

In *db/db* mice, in contrast, canagliflozin affected neither the rectal temperature on days 3, 6, and 10 (Fig. 5A). In telemetry experiments, canagliflozin failed to affect the body core temperature at 09:30 on days 1–10 (Fig. 5B). Notably, body core temperature did not show significant circadian change in *db/db* mice and canagliflozin had no effect on it at 3:30, 9:30, 15:30, and 21:30 on day 6 (Fig. 5C). Likewise, canagliflozin had little effect on locomotor activity at 09:30 on days 1–10 (Fig. 5D). Locomotor activity did not show significant circadian change and canagliflozin had little effect on it at 3:30, 9:30, 15:30, and 21:30 on day 6 (Fig. 5E).

## Discussion

**Fig. 6:**
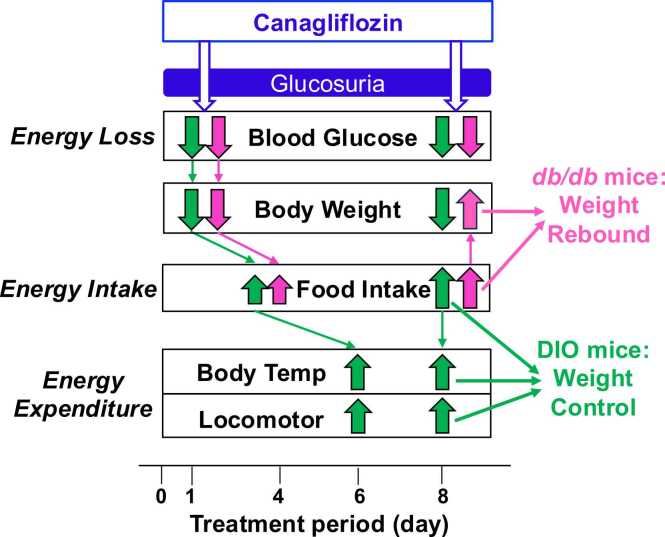
Proposed cascade for the action of canagliflozin on energy balance and weight outcome in DIO and *db/db* mice. Canagliflozin induces rapid glucose loss and weight reduction, followed by continuous increase in food intake in both DIO and *db/db* mice. This continuous increase in energy (food) intake leads to weight rebound in *db/db* mice. In DIO mice, canagliflozin subsequently induces circadian rises in body temperature and locomotor activity, exhibiting promoted energy expenditure. The elevated energy (food) intake and promoted energy expenditure may be balanced to maintain the initially lowered weight via glucosuria, achieving sustained weight loss.

To explore whether physical/metabolic states can alter the weight-lowering ability of SGLT2 inhibitors, we studied the effects of canagliflozin in two weight-matched obese mouse models with different metabolic states: DIO mice and *db/db* mice. Canagliflozin lowered blood glucose and weight on day 1 of treatment in two models. This initial weight reduction was further progressed in DIO mice but reversed toward gain in *db/db* mice (Fig. 6). Cumulative food intake and/or daily food intake started to increase from day 4, reaching significant levels on day 8 and later in DIO mice and on days 5, 9 and later in *db/db* mice. These time courses suggest that in *db/db* mice the increased food intake may drive weight rebound on day 6 and later. Remarkably, in DIO mice, canagliflozin elevated body temperature and locomotor activity from day 6: these energy consuming activities may counteract the elevated food intake to maintain the weight loss in DIO mice. This study demonstrates that physical/metabolic states, independent of social/psychological factors, influence the weight outcome of treatment with canagliflozin.

The rebound weight gain in *db/db* mice observed in this study appears not to be consistent with previous studies reporting reduced visceral adipose tissue in *db/db* mice [27] and reduced body weight gain in Zucker diabetic fatty (ZDF) rats treated with canagliflozin [28]. The difference between the current and previous reports could be due to differences in the study design, animal and treatment conditions. However, this issue remains to be addressed by future studies.

In this study, the canagliflozin-induced glucosuria (energy loss) and initial weight reduction (reduced energy storage) were followed by elevated eating behavior (elevated energy intake) in both DIO and *db/db* mice, possibly reflecting a homeostatic response (Fig. 6). In *db/db* mice, the elevated energy intake may overwhelm the energy loss via glucosuria to drive weight gain. Of note, in DIO mice, canagliflozin raised body temperature and locomotor activity (elevated energy expenditure). The elevated energy expenditure may balance the elevated energy intake, achieving a new energy equilibrium, in which the initially lowered weight is maintained (Fig. 6). Our results suggest that canagliflozin can reset the weight set point [29], [30] to a lower level than the original in DIO mice.

Canagliflozin elevated body temperature and locomotor activity selectively at 09:30 in DIO mice, suggesting that their responses to canagliflozin are controlled by a common circadian rhythm-dependent system. This possibly involves the sympathetic nerve, the system regulating body temperature [31], [32], locomotor activity [33] and circadian rhythm [34]. Furthermore, it has been reported that the sympathetic nerve is implicated in the action of SGLT2 inhibitors [35], [36]. Our finding fits with previous reports that SGLT2 inhibitors alter the circadian rhythms of body temperature [37] and locomotor activities in rats [36].

Intriguingly, we found that canagliflozin failed to elevate body temperature and locomotor activity in leptin receptor-deficient *db/db* mice. Hence, the system to elevate body temperature and locomotor activity may not function properly in the absolute absence of leptin signaling. It has been documented that leptin regulates body temperature and thermogenesis [38], [39], [40] and increases locomotor activity in a circadian-dependent manner [41], [42]. Notably, leptin is implicated in the set point of weight [30]. Taken together, leptin may serve as a factor to support canagliflozin-induced circadian rises in body temperature and locomotor activity. In addition to the leptin-leptin receptor axis, the gut hormones [43] such as ghrelin [44] that regulate food intake, body weight, body temperature, locomotor activity and circadian rhythm could also be implicated in the body’s responses to canagliflozin. However, further studies are definitely needed to elucidate the mechanisms for circadian rises in body temperature and locomotor activity under canagliflozin treatment.

In this study in mice treated with canagliflozin, weight was well controlled in DIO mice but rebounded in *db/db* mice, a model characterized by leptin unresponsiveness and severe insulin resistance [45]. This suggests that the degree of leptin resistance and insulin resistance in subjects could predict the weight outcome. Severe insulin and leptin resistances occur in people with morbid obesity. Monitoring body mass index (BMI), plasma insulin and leptin levels could help in selecting sustained responders to SGLT2 inhibitors for weight control.

## Conclusion

The present study explored whether physical/metabolic states, independent of social/psychological factors, influence weight outcome of therapy with SGLT2 inhibitors, by using obese models with distinct metabolic states, DIO mice and *db/db* mice. We found canagliflozin induces rapid glucose loss and weight reduction, followed by continuous increase in food intake in both models, possibly reflecting the body’s counter response to reduced energy storage [46], [47], [48]. This continuous increase in energy (food) intake leads to weight rebound in *db/db* mice. In DIO mice, in contrast, canagliflozin subsequently induces circadian rises in body temperature and locomotor activity, indicative of promoted energy expenditure. The elevated energy (food) intake and promoted energy expenditure may be balanced to maintain the initially lowered weight via glucosuria, achieving sustained weight loss (Fig. 6). This study indicates that physical/metabolic states influence weight outcome of therapy with SGLT2 inhibitors.

## Limitation

This study was conducted only in male mice. Clinical studies showed that canagliflozin gender-irrespectively lowers hemoglobin A1c and body weight [14] and reduces cardiovascular and renal events in type 2 diabetes [49], [50], indicating the sex-independent action of this drug: these reports support that the results of the present study may not be sex-limited. However, considering reported sex differences in metabolism and feeding, possible sex differences in effects and involvement of sex hormones remain to be studied. The treatment period was 10–16 days. This period appears to be reasonable in the light of mouse lifespan but much shorter than that for obesity treatment in humans. Hence, clinical extrapolation of the present results requires careful consideration.

## CRediT authorship contribution statement

**Yutaka Seino:** Supervision. **Toshihiko Yada:** Writing – original draft, Investigation, Funding acquisition, Conceptualization. **Daisuke Yabe:** Supervision. **Masanori Nakata:** Methodology. **Yusaku Iwasaki:** Methodology. **Abilkhair Yussupov:** Investigation. **Nazymgul Kulzhanova:** Investigation. **Yermek Rakhat:** Investigation. **Seiya Banno:** Investigation. **Dauren Zhantleu:** Investigation. **Wanxin Han:** Investigation. **Lei Wang:** Writing – original draft, Methodology, Investigation.

## Funding

This work was supported by Grant-in-Aid for Scientific Research (C) (25K10163) from Japan Society for the Promotion of Science (JSPS), COMIT Collaborative Research 2024, and Japan Association for Diabetes Education and Care (JADEC) 2024 to T.Y.

## Declaration of Competing Interest

The authors declare the following financial interests/personal relationships which may be considered as potential competing interests: Toshihiko Yada reports financial support was provided by Tanabe Pharma Corporation. If there are other authors, they declare that they have no known competing financial interests or personal relationships that could have appeared to influence the work reported in this paper.

## Data Availability

Data will be made available on reasonable request.

## References

[1] Ali A., Bain S., Hicks D., Newland Jones P., Patel D.C., Evans M., As part of The Improving Diabetes Steering C (2019). SGLT2 Inhibitors: Cardiovascular Benefits Beyond HbA1c-Translating Evidence into Practice. Diabetes Ther.

[2] Williams D.M., Nawaz A., Evans M. (2020). Renal outcomes in Type 2 Diabetes: a review of cardiovascular and renal outcome trials. Diabetes Ther..

[3] Musso G., Gambino R., Cassader M., Pagano G. (2012). A novel approach to control hyperglycemia in type 2 diabetes: sodium glucose co-transport (SGLT) inhibitors: systematic review and meta-analysis of randomized trials. Ann Med.

[4] Brown E., Heerspink H.J.L., Cuthbertson D.J., Wilding J.P.H. (2021). SGLT2 inhibitors and GLP-1 receptor agonists: established and emerging indications. Lancet.

[5] List J.F., Woo V., Morales E., Tang W., Fiedorek F.T. (2009). Sodium-glucose cotransport inhibition with dapagliflozin in type 2 diabetes. Diabetes Care.

[6] Brown E., Wilding J.P.H., Barber T.M., Alam U., Cuthbertson D.J. (2019). Weight loss variability with SGLT2 inhibitors and GLP-1 receptor agonists in type 2 diabetes mellitus and obesity: mechanistic possibilities. Obes Rev.

[7] Pereira M.J., Eriksson J.W. (2019). Emerging role of SGLT-2 inhibitors for the treatment of obesity. Drugs.

[8] Ferrannini G., Hach T., Crowe S., Sanghvi A., Hall K.D., Ferrannini E. (2015). Energy balance after sodium-glucose cotransporter 2 inhibition. Diabetes Care.

[9] Thewjitcharoen Y., Yenseung N., Malidaeng A., Nakasatien S., Chotwanvirat P., Krittiyawong S. (2017). Effectiveness of long-term treatment with SGLT2 inhibitors: real-world evidence from a specialized diabetes center. Diabetol Metab Syndr.

[10] Cain A.S., Bardone-Cone A.M., Abramson L.Y., Vohs K.D., Joiner T.E. (2010). Prospectively predicting dietary restraint: the role of interpersonal self-efficacy, weight/shape self-efficacy, and interpersonal stress. Int J. Eat Disord.

[11] Alqahtani R.M., Alhazmi A. (2025). Association between cognitive restraint, emotional eating, uncontrolled eating, and body mass index among health care professionals. Sci Rep.

[12] Tomiyama A.J. (2019). Stress and Obesity. Annu Rev Psychol..

[13] Teixeira P.J., Going S.B., Sardinha L.B., Lohman T.G. (2005). A review of psychosocial pre-treatment predictors of weight control. Obes Rev.

[14] Blonde L., Woo V., Mathieu C., Yee J., Vijapurkar U., Canovatchel W. (2015). Achievement of treatment goals with canagliflozin in patients with type 2 diabetes mellitus: a pooled analysis of randomized controlled trials. Curr Med Res Opin.

[15] Hanlon P., Butterly E., Wei L., Wightman H., Almazam S.A.M., Alsallumi K. (2025). Age and sex differences in efficacy of treatments for Type 2 Diabetes: a network meta-analysis. JAMA.

[16] Guo C., Dou J.H., Guo F.S., Zhao S., Wu R.Y., Hu Y.W. (2025). Sex-consistent outcomes in SGLT2 inhibitor-treated acute myocardial infarction patients: a real-world study. Eur J Med Res.

[17] Mori K., Saito R., Nakamaru Y., Shimizu M., Yamazaki H. (2016). Physiologically based pharmacokinetic-pharmacodynamic modeling to predict concentrations and actions of sodium-dependent glucose transporter 2 inhibitor canagliflozin in human intestines and renal tubules. Biopharm Drug Dispos.

[18] Kuriyama C., Xu J.Z., Lee S.P., Qi J., Kimata H., Kakimoto T. (2014). Analysis of the effect of canagliflozin on renal glucose reabsorption and progression of hyperglycemia in zucker diabetic Fatty rats. J Pharm Exp Ther.

[19] MacDonald T.L., Pattamaprapanont P., Cooney E.M., Nava R.C., Mitri J., Hafida S. (2022). Canagliflozin prevents hyperglycemia-associated muscle extracellular matrix accumulation and improves the adaptive response to aerobic exercise. Diabetes.

[20] Kawarasaki S., Sawazaki H., Iijima H., Ng S.P., Kwon J., Mohri S. (2020). Comparative Analysis of the Preventive Effects of Canagliflozin, a Sodium-Glucose Co-Transporter-2 Inhibitor, on Body Weight Gain Between Oral Gavage and Dietary Administration by Focusing on Fatty Acid Metabolism. Diab Metab Syndr Obes.

[21] Hasegawa S., Yamashita R., Nakagawa Y., Miyatake K., Katagiri H., Nakamura T. (2024). A novel methodology utilizing microchip implants to monitor individual activity and body temperature for assessing knee pain in group-housed rats. Sci Rep.

[22] Yoshizawa T., Shimada S., Takizawa Y., Makino T., Kanada Y., Ito Y. (2019). Continuous measurement of locomotor activity during convalescence and acclimation in group-housed rats. Exp Anim.

[23] Takano M., Okada T., Shioda K., Yonekawa C., Suda S. (2024). Risperidone suppresses caffeine-induced hyperthermia and hyperactivity in rats. Neurosci Lett.

[24] Refinetti R. (2020). Circadian rhythmicity of body temperature and metabolism. Temperature.

[25] Miyake T., Inoue Y., Maekawa Y., Doi M. (2024). Circadian clock and body temperature. Adv Exp Med Biol.

[26] Linhares S.S.G., Meurer Y., Aquino A., Camara D.A., Brandao L.E.M., Dierschnabel A.L. (2022). Effects of prenatal exposure to fluoxetine on circadian rhythmicity in the locomotor activity and neuropeptide Y and 5-HT expression in male and female adult Wistar rats. Int J Dev Neurosci.

[27] Qu J., Tian L., Zhang M., Sun B., Chen L. (2024). SGLT2 inhibitor canagliflozin reduces visceral adipose tissue in db/db mice by modulating AMPK/KLF4 signaling and regulating mitochondrial dynamics to induce browning. Mol Cell Endocrinol.

[28] Liang Y., Arakawa K., Ueta K., Matsushita Y., Kuriyama C., Martin T. (2012). Effect of canagliflozin on renal threshold for glucose, glycemia, and body weight in normal and diabetic animal models. PLoS One.

[29] Farias M.M., Cuevas A.M., Rodriguez F. (2011). Set-point theory and obesity. Metab Syndr Relat Disord.

[30] Speakman J.R., Levitsky D.A., Allison D.B., Bray M.S., de Castro J.M., Clegg D.J. (2011). Set points, settling points and some alternative models: theoretical options to understand how genes and environments combine to regulate body adiposity. Dis Model Mech.

[31] Nakamura K. (2011). Central circuitries for body temperature regulation and fever. Am J Physiol Regul Integr Comp Physiol.

[32] Nakamura K., Morrison S.F. (2022). Central sympathetic network for thermoregulatory responses to psychological stress. Auton Neurosci.

[33] Cowley K.C. (2018). A new conceptual framework for the integrated neural control of locomotor and sympathetic function: implications for exercise after spinal cord injury. Appl Physiol Nutr Metab.

[34] Hou T., Chacon A.N., Su W., Katsumata Y., Guo Z., Gong M.C. (2022). Role of sympathetic pathway in light-phase time-restricted feeding-induced blood pressure circadian rhythm alteration. Front Nutr.

[35] Spallone V., Valensi P. (2021). SGLT2 inhibitors and the autonomic nervous system in diabetes: a promising challenge to better understand multiple target improvement. Diabetes Metab.

[36] Rahman A., Fujisawa Y., Nakano D., Hitomi H., Nishiyama A. (2017). Effect of a selective SGLT2 inhibitor, luseogliflozin, on circadian rhythm of sympathetic nervous function and locomotor activities in metabolic syndrome rats. Clin Exp Pharmacol Physiol.

[37] Iuchi H., Sakamoto M., Matsutani D., Suzuki H., Kayama Y., Takeda N. (2017). Time-dependent effects of ipragliflozin on behaviour and energy homeostasis in normal and type 2 diabetic rats: continuous glucose telemetry analysis. Sci Rep.

[38] Deem J.D., Muta K., Ogimoto K., Nelson J.T., Velasco K.R., Kaiyala K.J. (2018). Leptin regulation of core body temperature involves mechanisms independent of the thyroid axis. Am J Physiol Endocrinol Metab.

[39] Genchi V.A., D'Oria R., Palma G., Caccioppoli C., Cignarelli A., Natalicchio A. (2021). Impaired leptin signalling in obesity: is leptin a new thermolipokine?. Int J Mol Sci.

[40] Fischer A.W., Cannon B., Nedergaard J. (2020). Leptin: is it thermogenic?. Endocr Rev.

[41] Vivas Y., Azpeleta C., Feliciano A., Velarde E., Isorna E., Delgado M.J. (2011). Time-dependent effects of leptin on food intake and locomotor activity in goldfish. Peptides.

[42] Ribeiro A.C., Ceccarini G., Dupre C., Friedman J.M., Pfaff D.W., Mark A.L. (2011). Contrasting effects of leptin on food anticipatory and total locomotor activity. PLoS One.

[43] Lee W.J., Chen C.Y., Chong K., Lee Y.C., Chen S.C., Lee S.D. (2011). Changes in postprandial gut hormones after metabolic surgery: a comparison of gastric bypass and sleeve gastrectomy. Surg Obes Relat Dis.

[44] Chen C.Y., Fujimiya M., Laviano A., Chang F.Y., Lin H.C., Lee S.D. (2010). Modulation of ingestive behavior and gastrointestinal motility by ghrelin in diabetic animals and humans. J Chin Med Assoc.

[45] Bahary N., Leibel R.L., Joseph L., Friedman J.M. (1990). Molecular mapping of the mouse db mutation. Proc Natl Acad Sci USA.

[46] Sumithran P., Prendergast L.A., Delbridge E., Purcell K., Shulkes A., Kriketos A. (2011). Long-term persistence of hormonal adaptations to weight loss. N Engl J Med.

[47] Polidori D., Sanghvi A., Seeley R.J., Hall K.D. (2016). How strongly does appetite counter weight loss? Quantification of the feedback control of human energy intake. Obesity.

[48] Hintze L.J., Mahmoodianfard S., Auguste C.B., Doucet E. (2017). Weight loss and appetite control in women. Curr Obes Rep.

[49] Neal B., Perkovic V., Mahaffey K.W., de Zeeuw D., Fulcher G., Erondu N., Group CPC (2017). Canagliflozin and cardiovascular and renal events in Type 2 diabetes. N Engl J Med.

[50] Perkovic V., Jardine M.J., Neal B., Bompoint S., Heerspink H.J.L., Charytan D.M., Investigators CT (2019). Canagliflozin and Renal Outcomes in Type 2 Diabetes and Nephropathy. N Engl J Med.

